# OnabotulinumtoxinA Treatment for Masseter Muscle Prominence: 6-Month Safety and Efficacy Results, Including Patient-Reported Outcomes, From a Phase 3, Randomized, Placebo-Controlled, Multiregional Trial

**DOI:** 10.1093/asj/sjaf204

**Published:** 2025-10-15

**Authors:** Jiaming Sun, Lei Wang, Yan Wu, Shannon Humphrey, Shyi-Gen Chen, William McGillivray, Shu-Hung Huang, Yuguang Zhang, Grace Pan, Catherine Foley, Elisabeth Lee, Joan-En Chang-Lin, Donna L Faletto, Vaishali Patel, Beta Bowen, Mitchell F Brin

## Abstract

**Background:**

Masseter muscle prominence (MMP) may be aesthetically bothersome to some individuals, leading them to seek treatment for a slimmer lower face.

**Objectives:**

The aim of this study was to evaluate the safety and efficacy of onabotulinumtoxinA for the treatment of MMP, including patient-reported outcomes (PROs).

**Methods:**

This was a prospective, multicenter trial including a randomized, double-blind, placebo-controlled period (Days 1−180) in which adults rated Grade 4 or 5 (marked/very marked) on the investigator-assessed MMP Scale (MMPS) were randomized to onabotulinumtoxinA 72 U or placebo. Efficacy endpoints were assessed at Day 90. The primary endpoint was ≥2-grade improvement from baseline on the investigator-assessed MMPS. Secondary endpoints included achieving Grade ≤3 on the MMPS and participant-assessed MMPS—Participant (MMPS-P), ≥2-grade improvement on the participant-assessed MMPS-P, and change from baseline in lower-facial width. Outcomes were assessed using validated measures. Adverse events (AEs) were monitored.

**Results:**

Of 376 enrolled participants (onabotulinumtoxinA, *n* = 283; placebo, *n* = 93), 310 (82.4%) completed the study. At Day 90, a greater proportion of onabotulinumtoxinA-treated participants vs placebo achieved MMPS ≥2-grade improvement (51.2% vs 2.2%, *P* < .0001), and more onabotulinumtoxinA-treated participants vs placebo achieved the secondary endpoints (all *P* < .0001), with a mean lower-facial width reduction of −5.24 mm for onabotulinumtoxinA vs −0.04 mm for placebo (*P* < .0001). Participants reported benefits for onabotulinumtoxinA vs placebo in self-perceived change in MMP, treatment satisfaction, and psychosocial impact. Improvements were sustained through Day 180. Most AEs were mild, nonserious, and resolved.

**Conclusions:**

OnabotulinumtoxinA effectively reduced the appearance of MMP and improved PROs, with effects lasting up to 6 months and a favorable safety profile.

**Level of Evidence: 1 (Therapeutic):**

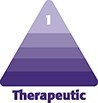

Masseter muscle prominence (MMP) is a condition characterized by enlargement of the masseter muscles, resulting in a square or trapezoidal lower-facial shape with a prominent mandibular angle.^1,2^ MMP may be considered aesthetically undesirable and bothersome in many cultures, including among Asian individuals.^3^ Affected individuals may experience negative psychological effects because of a perceived unattractive facial appearance, including emotional, social, relationship, and work impacts.^4-6^ Thus, some individuals with MMP may seek treatment to achieve a more aesthetically pleasing, slimmer lower face.^2,3,7^

Historically, cosmetic reduction of the masseter muscle has been accomplished through invasive treatment modalities, including surgical resection of the masseter muscle and ostectomy of the mandibular angle.^1,8^ These procedures may be associated with complications, such as injury to facial blood vessels or nerves, infection, scarring, and general anesthesia–related risks.^1,9^ OnabotulinumtoxinA (Botox Cosmetic; Allergan Aesthetics, an AbbVie company, Irvine, CA) is an intramuscular neuromodulator that causes chemical denervation and temporary muscle-activity reduction in the treated area.^10^ It has been utilized off-label for more than 20 years as a less-invasive option to reduce MMP, with multiple small studies and case reports showing that treatment may result in a more ovoid lower-facial shape.^2,3,7,11-14^ More recently, well-controlled clinical studies have established more clearly the safety and efficacy of onabotulinumtoxinA to treat MMP. From the results of a 12-month, randomized, double-blind, placebo-controlled, multicenter, dose-ranging (24, 48, 72, and 96 U), Phase 2 study in Australia, Taiwan, and Canada (NCT02010775), the authors demonstrated that 1 or 2 treatments of onabotulinumtoxinA were effective and well tolerated for reducing lower-facial volume in participants (*n* = 187) with bilateral MMP over 1 year.^15^

Given the critical importance of the patient's treatment perspective, the authors of the Phase 2 study (NCT02010775) included patient-reported outcome (PRO)-based endpoints as a key component of the study design.^16,17^ Patient-centric data were collected using MMP-specific, de novo PRO measures in addition to conventional efficacy data, such as lower-facial volume. After onabotulinumtoxinA treatment, participants reported improvements in their MMP symptoms and in the impacts of MMP. Patients who received onabotulinumtoxinA treatment reported greater treatment satisfaction than those who received placebo.

Here, findings are presented from a pivotal Phase 3 clinical trial conducted in China, Canada, and Taiwan to evaluate efficacy, safety, and patient-reported impact of onabotulinumtoxinA treatment for MMP. This study built on Phase 2 results and further assessed onabotulinumtoxinA 72 U treatment outcomes.

## METHODS

### Study Design and Participants

This 18-month, Phase 3 study was conducted at 21 sites in China, Canada, and Taiwan from August 2019 to November 2022. The study consisted of a double-blind, placebo-controlled (DBPC) period (Days 1-180; Period 1) and an open-label period (Days 180-540; Period 2; Supplemental Figure 1). The authors of this study enrolled adults (aged ≥18 years) with marked (Grade 4) or very marked (Grade 5) bilateral MMP on the investigator-assessed MMP Scale (MMPS) and pronounced (Grade 4) or very pronounced (Grade 5) MMP on the participant-assessed MMPS—Participant (MMPS-P). The MMPS is a validated 5-grade clinical scale for measuring MMP severity (1 = minimal, 2 = mild, 3 = moderate, 4 = marked, 5 = very marked) that encompasses both visual and palpable examination of the masseter muscle at rest and at jaw-clenched states. The MMPS-P is a validated 5-grade PRO measure for assessing MMP severity (1 = not at all pronounced, 2 = mildly pronounced, 3 = moderately pronounced, 4 = pronounced, 5 = very pronounced) whereby participants assess the size and shape of their lower face. Participants were excluded from the study if they had any history of conditions, treatments, or procedures that could interfere with normal chewing and jaw clenching or assessment of MMP, or if they had received botulinum toxin treatments to the masseter muscle or lower face at any time, or to any other part of the body within 6 months before Day 1. To avoid confounding the efficacy assessments, participants were required to maintain a stable weight (within 10% of baseline body weight) for the duration of the study.

In the DBPC period, participants were randomized 3:1 to receive a single treatment with onabotulinumtoxinA 72 U (6 injections [3 per masseter muscle] of 12 U [0.3 mL] each) or placebo on Day 1 (treatment Cycle 1). In the open-label period, all onabotulinumtoxinA- and placebo-treated participants who met re-treatment criteria (bilateral Grade 4 or 5 investigator-assessed MMP, not pregnant, and at least 3 months elapsed since previous study treatment) on or after the Day 180 visit received up to 2 onabotulinumtoxinA treatments. The results presented are for the DBPC period.

The study was approved by an IRB (Advarra, Inc, Aurora, ON, Canada) and conducted in accordance with the International Council for Harmonisation of Technical Requirements for Pharmaceuticals for Human Use guidelines, applicable regulations, and guidelines governing clinical study conduct and ethical principles that have their origin in the Declaration of Helsinki. All participants or their legally authorized representatives provided written informed consent.

### Treatment Administration

Injections were administered by a standardized protocol where the investigator first drew a line from the corner of the mouth to a point at the inferior border of the ear. The participant then maximally clenched their jaw, and the investigator outlined the area of maximal bulge of the masseter muscle (located below the drawn line and posterior to the risorius and anterior to the parotid gland). The bulkiest point of the masseter muscle was marked as the first injection site, and 2 additional sites spaced ∼1 cm apart within the treatment area were selected. The needle was inserted perpendicularly to the full depth of the muscle, and the injected volume was distributed within the deeper and more superficial muscle layers by withdrawing the needle throughout the injection. This same procedure was followed for the contralateral masseter muscle. The onabotulinumtoxinA dose was based on previously published Phase 2 safety and efficacy results.^15^

### Study Assessments

#### Efficacy Assessment Using Clinician-Reported Outcomes, Patient-Reported Outcomes (PROs), and Images

Instruments used in study assessments are summarized in Supplemental Table 1. The primary efficacy endpoint was the achievement of ≥2-grade improvement from baseline on the MMPS at Day 90. Secondary efficacy endpoints evaluated at Day 90 included achievement of Grade ≤3 on investigator-assessed MMPS and participant-assessed MMPS-P, achievement of ≥2-grade improvement from baseline on MMPS-P, change from baseline in lower-facial width calculated from standardized images using the VECTRA M3 3-dimensional imaging system (Canfield Scientific, Parsippany-Troy Hills, NJ), and achievement of Participant Self-Assessment of Change (PSAC) Grade ≥1 (at least minimally improved from baseline). Facial width was measured as the width of the face at the level of the stomion (the midline point at the junction of the upper and lower lip vermillion). The PSAC is an assessment-of-change scale adapted for use with MMP, in which participants assessed the change in their lower-face shape from before to after the study intervention (3 = much improved, 2 = moderately improved, 1 = minimally improved, 0 = no change, −1 = minimally worse, −2 = moderately worse, −3 = much worse).

Additional endpoints were based on the following de novo, validated PRO measures: Lower Facial Shape Questionnaire (LFSQ)—Treatment Satisfaction Assessment (LFSQ-TXSAT, follow-up version), Participant Global Impression of Bother (PGIB), and LFSQ—Impact Assessment (LFSQ-IA). PRO measures were developed, validated, translated, and implemented in accordance with FDA PRO Guidance and other international regulatory and professional society standards.^18-22^ The LFSQ-TXSAT (follow-up version) was used to assess participants’ satisfaction with the effect of treatment on their lower face appearance using a 5-point scale ranging from “very satisfied” to “very dissatisfied,” whereas the PGIB assessed how bothered participants were by the appearance of their lower face on a 5-point scale ranging from “not at all bothered” to “extremely bothered.” The LSFQ-IA was used to assess the psychosocial impact associated with lower face appearance (ie, feel less attractive, self-conscious, confident, sad, embarrassed, and outgoing around others) and is reflected as a summary score ranging from 0 to 24, with a lower score indicating a decrease in unfavorable appearance-related psychosocial impact; a decrease from baseline signifies improvement.

#### Safety

Adverse events (AEs) were monitored throughout the study, as well as vital signs, and there was screening for temporomandibular joint disorder.

### Statistical Analysis

Efficacy analyses were conducted in the modified intent-to-treat (mITT) population, defined as all randomized participants with ≥1 postbaseline MMPS assessment. Safety analyses were conducted in the safety population, defined as all treated participants. Based on a previous study with a placebo responder rate of 8%, it was determined that ∼360 participants (270 onabotulinumtoxinA and 90 control) would provide >90% power to detect a ≥18% difference in MMPS response rate between onabotulinumtoxinA 72 U and control using a 2-sided Mantel–Haenszel test at a 5% significance level.^15^ All statistical tests were 2-sided hypothesis tests performed at the 5% significance level. Between-treatment comparisons for binary endpoints were analyzed using the Cochran–Mantel–Haenszel test stratified by baseline MMPS grade (4 or 5). Between-treatment comparisons for continuous endpoints (change from baseline in lower-facial width) were analyzed using analysis of covariance with treatment group and investigator sites as factors, and baseline MMPS grade and applicable baseline scores as covariates. For the primary and secondary endpoints, missing values up to Day 180 were imputed using multiple imputations. Statistical significance was defined as *P* ≤ .05.

## RESULTS

### Participants

Of 377 enrolled and randomized participants, 376 were treated (onabotulinumtoxinA 72 U, *n* = 283; placebo, *n* = 93), 370 had ≥1 postbaseline MMPS assessment, and 341 (90.5%) completed treatment Cycle 1 (Table 1); 1 participant was randomized in error and not treated. Reasons for discontinuation in the DBPC period included withdrawal by participant (*n* = 26), loss to follow-up (*n* = 3), protocol deviation (*n* = 3), AE (*n* = 2), and COVID-19–related reasons (*n* = 1). Participants who completed the study (*n* = 310, 82.2%) were followed for 18 months. The mean age was 31.4 years (range, 19-70 years), 88.1% were female (*n* = 326 females; *n* = 44 males), and 82.4% were Chinese. MMP for all participants was graded either marked (Grade 4; 63.7%) or very marked (Grade 5; 36.3%) on the investigator-assessed MMPS and pronounced (Grade 4; 62.2%) or very pronounced (Grade 5; 37.8%) on the participant-assessed MMPS-P. Responses to the baseline version of the LFSQ-TXSAT, which measures treatment expectations, indicated that most participants expected the study treatment to improve their lower-facial appearance “quite a bit” (55.7%), followed by “somewhat” (21.4%) and “extremely” (19.5%).

**Table 1.** Demographics and Baseline Characteristics (mITT Population)

	Placebo (*n* = 92)	OnabotA (*n* = 278)	Total (*n* = 370)
Age, mean years (SD)	31.0 (8.7)	31.5 (8.4)	31.4 (8.4)
Range	19-60	19-70	19-70
Sex, *n* (%)			
Female	82 (89.1)	244 (87.8)	326 (88.1)
Race, *n* (%)			
Asian	76 (82.6)	247 (88.8)	323 (87.3)
Chinese	73 (79.3)	232 (83.5)	305 (82.4)
White	15 (16.3)	29 (10.4)	44 (11.9)
Other	1 (1.1)	2 (0.7)	3 (0.8)
MMPS, *n* (%)			
Grade 4: marked	59 (64.1)	177 (63.7)	236 (63.8)
Grade 5: very marked	33 (35.9)	101 (36.3)	134 (36.2)
MMPS-P, *n* (%)			
Grade 4: pronounced	57 (62.0)	173 (62.2)	230 (62.2)
Grade 5: very pronounced	35 (38.0)	105 (37.8)	140 (37.8)
LFSQ-TXSAT (baseline version)^a^, *n* (%)			
Not at all	1 (1.1)	1 (0.4)	2 (0.5)
A little	2 (2.2)	9 (3.2)	11 (3.0)
Somewhat	28 (30.4)	51 (18.3)	79 (21.4)
Quite a bit	45 (48.9)	161 (57.9)	206 (55.7)
Extremely	16 (17.4)	56 (20.1)	72 (19.5)

LFSQ-TXSAT, Lower Facial Shape Questionnaire—Treatment Satisfaction Assessment; mITT, modified intent-to-treat; MMPS, Masseter Muscle Prominence Scale; MMPS-P, Masseter Muscle Prominence Scale—Participant; onabotA, onabotulinumtoxinA; SD, standard deviation.

^a^The LFSQ-TXSAT (baseline version) is a 1-item measure assessing participant expectations of study treatment to improve lower-facial appearance.

### Primary and Secondary Efficacy Endpoints Using Clinician-Reported Outcomes, PROs, and Images

**Figure 1.**
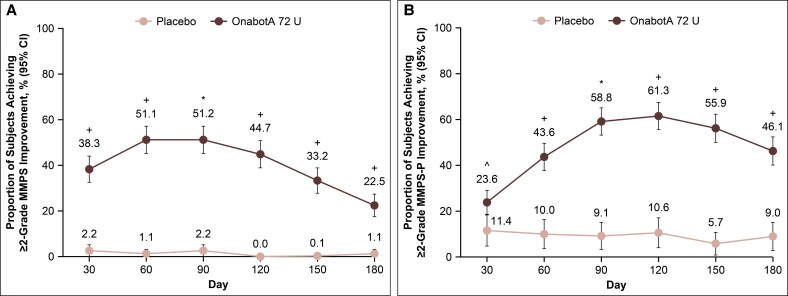
Proportion of participants achieving (A) ≥2-grade improvement from baseline on the investigator-assessed MMPS and (B) ≥2-grade improvement on the participant-assessed MMPS-P through Day 180. *P < .0001 vs placebo; ^^^nominal *P* < .02 vs placebo; ^+^nominal *P* < .0001 vs placebo. MMPS, Masseter Muscle Prominence Scale; MMPS-P, Masseter Muscle Prominence Scale—Participant; onabotA, onabotulinumtoxinA.

**Figure 2.**
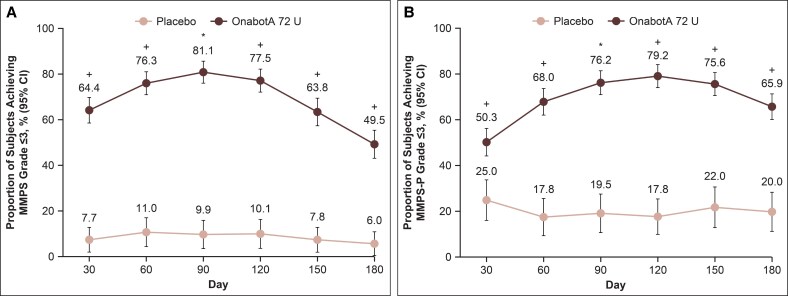
Proportion of participants achieving (A) Grade ≤3 on the investigator-assessed MMPS and (B) Grade ≤3 on the participant-assessed MMPS-P through Day 180. Error bars represent 95% CIs. *P < .0001 vs placebo; ^+^nominal *P* < .0001 vs placebo. MMPS, Masseter Muscle Prominence Scale; MMPS-P, Masseter Muscle Prominence Scale—Participant; onabotA, onabotulinumtoxinA.

The primary endpoint was met in the DBPC period, with 51.2% of onabotulinumtoxinA 72 U–treated participants compared with 2.2% of placebo-treated participants, demonstrating a ≥2-grade improvement from baseline on the investigator-assessed MMPS at Day 90 (*P* < .0001; Figure 1A). The proportion of participants achieving Grade ≤3 on the MMPS was also significantly greater for onabotulinumtoxinA vs placebo at Day 90 (81.1% vs 9.9%, *P* < .0001; Figure 2A). For both outcomes, significant benefit for onabotulinumtoxinA compared with placebo was observed as early as Day 30 (first time point assessed) and through Day 180.

Similar patterns were observed in the proportions of participants achieving a ≥2-grade improvement from baseline or Grade ≤3 on the participant-assessed MMPS-P at Day 90, both significantly greater for onabotulinumtoxinA vs placebo (≥2-grade improvement, 58.8% vs 9.1%; Grade ≤3, 76.2% vs 19.5%; *P* < .0001 for both; Figures 1B, 2B). Similarly, the pattern of significant benefit for onabotulinumtoxinA compared with placebo was observed as early as Day 30 (first time point assessed) and through Day 180 for both outcomes.

**Figure 3.**
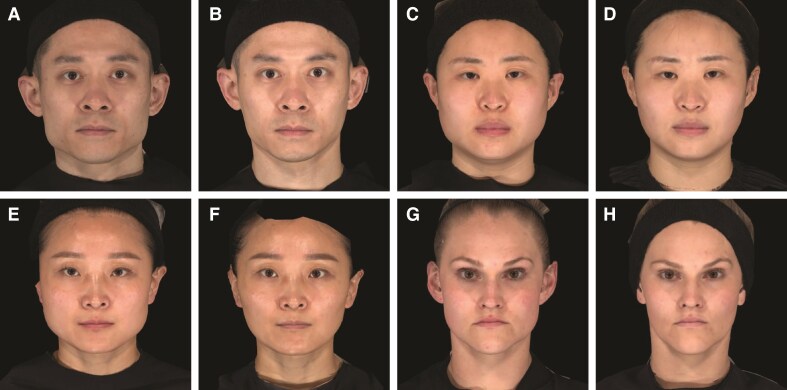
Representative photos of participants with ≥2-grade improvement on the investigator-assessed MMPS and participant-assessed MMPS-P before treatment and at Day 90 after treatment with onabotulinumtoxinA 72 U. All participants reported being “satisfied” or “very satisfied” at posttreatment time points. (A) A 43-year-old male patient from Taiwan before treatment (MMPS: left, 5; right, 5; MMPS-P: 5) and (B) at Day 90 (MMPS: left, 3; right, 3; MMPS-P: 2). (C) A 30-year-old female patient from China before treatment (MMPS: left, 4; right, 4; MMPS-P: 4) and (D) at Day 90 (MMPS: left, 3; right, 3; MMPS-P: 4). (E) A 40-year-old female patient from China before treatment (MMPS: left, 5; right, 5; MMPS-P: 5) and (F) at Day 90 (MMPS: left, 3; right, 3; MMPS-P: 3). (G) A 31-year-old female patient from Canada before treatment (MMPS: left, 4; right, 4; MMPS-P: 4) and (H) at Day 90 (MMPS: left, 2; right, 2; MMPS-P: 1). MMPS, Masseter Muscle Prominence Scale; MMPS-P, Masseter Muscle Prominence Scale—Participant.

**Figure 4.**
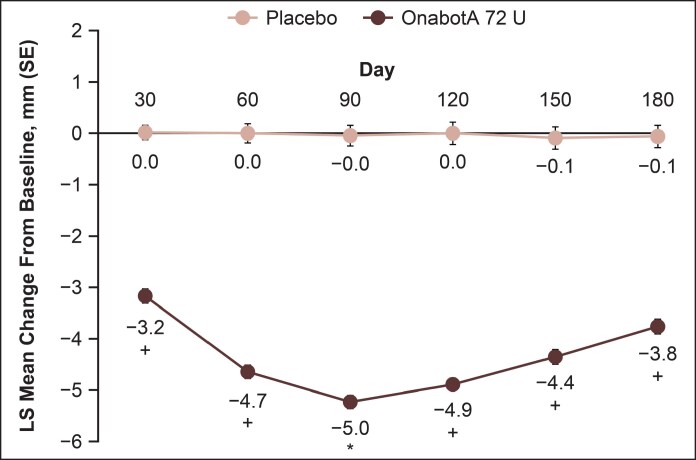
Least-squares mean change from baseline in lower-facial width through Day 180. *P < .0001 vs placebo; ^+^nominal *P* < .0001 vs placebo. OnabotA, onabotulinumtoxinA.

**Figure 5.**
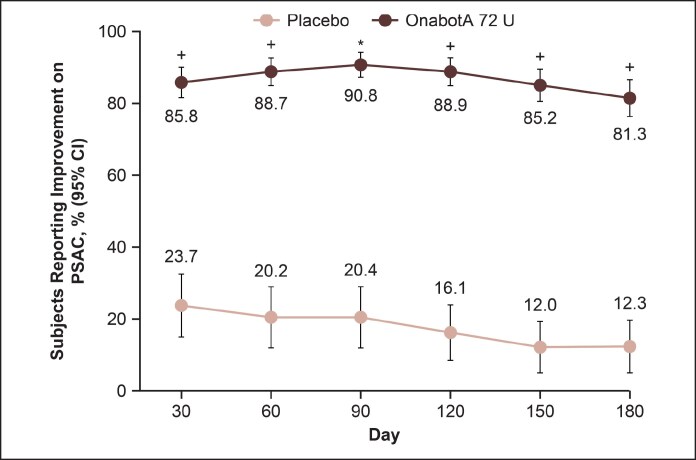
Participants reporting at least minimal improvement from baseline in lower facial shape on PSAC through Day 180. The PSAC assesses change in lower face shape on a 7-point scale ranging from “much worse” to “much improved.” *P < .0001 vs placebo. ^+^nominal *P* < .0001 vs placebo. OnabotA, onabotulinumtoxinA; PSAC, Participant Self-Assessment of Change.

Representative photographs of participants from Taiwan, China, and Canada with a ≥2-grade improvement from baseline on the investigator-assessed MMPS and participant-assessed MMPS-P at Day 90 are shown in Figure 3. All participants reported being “satisfied” or “very satisfied” at posttreatment time points. The mean reduction from baseline in lower-facial width at Day 90 was significantly higher in the onabotulinumtoxinA 72 U group compared with the placebo group (−5.24 vs −0.04 mm, *P* < .0001; Figure 4). Also, a significantly greater proportion of participants treated with onabotulinumtoxinA 72 U vs placebo achieved PSAC Grade ≥1 (at least minimally improved from baseline) at Day 90 (90.8% vs 20.4%, respectively; *P* < .0001). Responder rates with onabotulinumtoxinA 72 U remained high (>80%) and greater than placebo through Day 180 (Figure 5).

### Additional Efficacy Endpoints Assessment Using PROs

**Figure 6.**
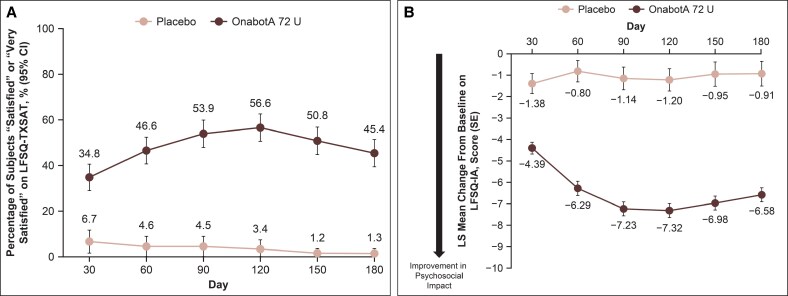
LFSQ endpoints through Day 180. (A) Participants reporting being “very satisfied” or “satisfied” with the effect of treatment on the LFSQ-TXSAT (follow-up version). The LFSQ-TXSAT follow-up version measures treatment satisfaction on a 5-point scale ranging from “very satisfied” to “very dissatisfied.” Nominal *P* < .0001 vs placebo at all time points. (B) Improvement in appearance-related psychosocial impact associated with MMP as assessed by the LFSQ-IA. Baseline mean (standard deviation) LFSQ-IA summary score: onabotA, 15.23 (5.91); placebo, 14.39 (5.50). The LFSQ-IA measures psychosocial impact associated with lower face appearance; range, 0-24; lower scores indicate less psychosocial impact and decreases from baseline signify improvement. Nominal *P* < .0001 vs placebo at all time points. LFSQ, Lower Facial Shape Questionnaire; LFSQ-IA, Lower Facial Shape Questionnaire—Impact Assessment; LFSQ-TXSAT, Lower Facial Shape Questionnaire—Treatment Satisfaction Assessment; LS, least squares; onabotA, onabotulinumtoxinA; SE, standard error of the mean.

The proportion of participants reporting on the LFSQ-TXSAT (follow-up version) that they were either “very satisfied” or “satisfied” with the treatment effect (ie, responders) was greater with onabotulinumtoxinA 72 U vs placebo at Day 90 (53.9% vs 4.5%, respectively; nominal *P* < .0001; Figure 6A). Responder rates remained greater with onabotulinumtoxinA 72 U than with placebo through Day 180 (nominal *P* < .0001).

At baseline, psychosocial impact associated with lower-facial appearance as indicated by mean (standard deviation) LFSQ-IA summary scores was 15.23 (5.91) for the onabotulinumtoxinA group and 14.39 (5.50) for the placebo group. Greater improvements in psychosocial impact were seen with onabotulinumtoxinA 72 U vs placebo at Day 90 (least squares mean decrease from baseline in LFSQ-IA summary score of −7.23 vs −1.14, respectively; nominal *P* < .0001; Figure 6B). Differences between treatment groups remained through Day 180 (nominal *P* < .0001).

**Figure 7.**
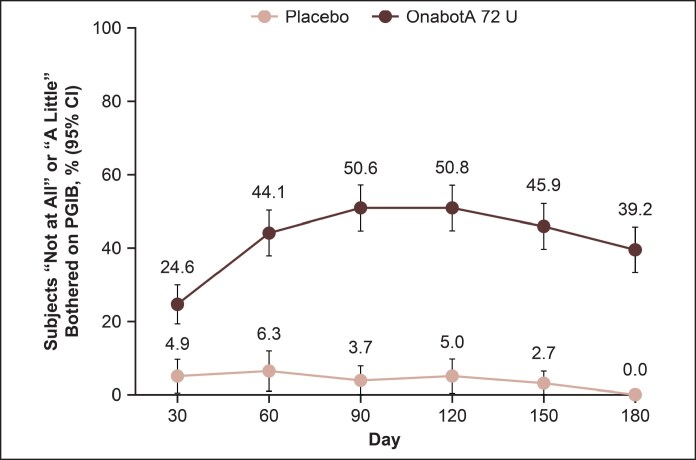
Participants reporting being “not at all bothered” or “a little bothered” after treatment on the PGIB through Day 180. PGIB measures level of bother associated with MMP on a 5-point scale, ranging from “not at all bothered” to “extremely bothered.” At baseline, 93.5% and 91.3% of participants in the onabotulinumtoxinA and placebo groups, respectively, reported being “somewhat bothered,” “a lot bothered,” or “extremely bothered” by the appearance of their lower face. Nominal *P* < .0001 vs placebo at all time points. OnabotA, onabotulinumtoxinA; PGIB, Participant Global Impression of Bother; MMP, Masseter Muscle Prominence Scale.

Median PGIB scores at baseline (onabotulinumtoxinA 72 U, 3.0; placebo, 3.0) indicated that participants were “a lot bothered” by the appearance of their lower face before treatment. Among participants who were at least “somewhat bothered” by the appearance of their lower face at baseline (>90% of the study population), the proportion reporting that they were “not at all bothered” or “a little bothered” at Day 90 was greater with onabotulinumtoxinA 72 U vs placebo (50.6% vs 3.7%, respectively; nominal *P* < .0001; Figure 7). These differences between groups persisted through Day 180.

### Safety

During the DBPC period, 44.5% of onabotulinumtoxinA-treated participants and 35.5% of placebo-treated participants reported at least 1 treatment-emergent AE (TEAE; Table 2). The most frequently reported TEAE was upper respiratory tract infection (4.9% for onabotulinumtoxinA vs 3.2% for placebo), followed by nasopharyngitis (4.6% vs 3.2%), mastication disorder (4.2% vs 0%), and injection-site pain (2.8% vs 2.2%). Two TEAEs leading to study discontinuation (post–acute COVID-19 syndrome and paradoxical masseter muscle bulging) were reported in the onabotulinumtoxinA group.

**Table 2.** Summary of Treatment-Emergent Adverse Events During the Double-Blind, Placebo-Controlled Period (Safety Population)

AE category, *n* (%)	Placebo (*n* = 93)	OnabotA (*n* = 283)
Any TEAE^a^	33 (35.5)	126 (44.5)
Upper respiratory tract infection	3 (3.2)	14 (4.9)
Nasopharyngitis	3 (3.2)	13 (4.6)
Mastication disorder	0	12 (4.2)
Injection-site pain	2 (2.2)	8 (2.8)
Acne	1 (1.1)	7 (2.5)
Injection-site discomfort	1 (1.1)	7 (2.5)
Pyrexia	1 (1.1)	7 (2.5)
Headache	2 (2.2)	6 (2.1)
Cough	1 (1.1)	6 (2.1)
Periodontitis	0	6 (2.1)
Treatment-related TEAE	3 (3.2)	34 (12.0)
Mastication disorder	0	10 (3.5)
Injection-site pain	2 (2.2)	8 (2.8)
Injection-site discomfort	0	7 (2.5)
Injection-site muscle weakness	0	4 (1.4)
Paradoxical masseter muscle bulging	0	6 (2.1)
Facial paresis	0	2 (0.7)
Headache	0	2 (0.7)
Muscular weakness	0	1 (0.4)
TEAE leading to discontinuation	0	2 (0.7)
Post-acute COVID-19 syndrome	0	1 (0.4)
Paradoxical masseter muscle bulging	0	1 (0.4)
Possible distant spread of toxin^b^	1 (1.1)^c^	8 (2.8)^d^

AE, adverse event; onabotA, onabotulinumtoxinA; TEAE, treatment-emergent adverse event.

^a^Events reported in >2% of participants are shown.

^b^All PDSOTs were medically adjudicated and found to be local events or unrelated to the toxin.

^c^Includes 1 participant reporting constipation.

^d^Includes 2 participants reporting facial paresis and 1 participant each reporting accommodation disorder, Bell's palsy, bradycardia, dry mouth, dyspnea, and muscular weakness.

Treatment-related AEs were reported during the DBPC period by 12.0% and 3.2% of participants in the onabotulinumtoxinA and placebo groups, respectively (Table 2). The most frequent were mastication disorder (3.5%, eg, masseter/jaw fatigue, masseter/jaw pain, and chewing weakness) and injection-site pain (2.8%) in those who received onabotulinumtoxinA. In addition, 9 participants (onabotulinumtoxinA, *n* = 8; placebo, *n* = 1) reported possible distant spread of toxin (PDSOT) events, which included 1 case of constipation in the placebo group, 2 cases of facial paresis in the onabotulinumtoxinA group, and 1 case each of accommodation disorder, Bell's palsy, bradycardia, dry mouth, dyspnea, and muscular weakness in the onabotulinumtoxinA group. All PDSOT events were mild in severity, required no intervention, and resolved. Upon adjudication, all events were local or unrelated to toxin.

## DISCUSSION

In this prospective, multicenter, Phase 3 study, a single treatment of onabotulinumtoxinA 72 U demonstrated significant improvement in MMP as determined by investigator- and participant-assessed measures (ie, improvement from baseline in MMPS and MMPS-P grades) as well as by objective lower-facial width measurements based on standardized images, with improvements in efficacy outcomes peaking around Day 90 and continuing through Day 180. AEs related to treatment with onabotulinumtoxinA 72 U were primarily local and consistent with the well-established safety profile of the toxin. These findings are consistent with those of a previous Phase 2 study, which demonstrated that 1 or 2 treatments of up to 96 U onabotulinumtoxinA were effective and well tolerated for reducing lower-facial volume in participants with bilateral MMP, with the peak facial-volume decrease occurring at Day 90 and sustained for up to 6 months.^15^

PROs are increasingly recognized as providing clinicians with valuable insights into how patients perceive the impact of their treatment. The present study demonstrated that participants with bilateral investigator-assessed marked or very marked MMP experienced multiple benefits within 90 days after receiving a single treatment of onabotulinumtoxinA 72 U. Greater proportions of onabotulinumtoxinA-treated participants vs placebo-treated participants reported that their lower-facial shape was at least minimally improved from baseline and that they were “very satisfied” or “satisfied” with the treatment effect. Participants also reported reduced levels of bother with the appearance of their lower face and greater improvements in appearance-related psychosocial impact after onabotulinumtoxinA treatment compared with placebo. These effects were maintained to 180 days posttreatment.

In this study, decreases in lower-facial width (an objective measure of change) were aligned with improvements in investigator-assessed MMP as measured by the MMPS, both peaking at Day 90. Interestingly, improvement in the participant-assessed MMPS-P scores peaked slightly later, at Day 120, and had a less pronounced drop-off toward Day 180 compared with objective and investigator-assessed measures. These findings suggest that the patient-perceived onabotulinumtoxinA treatment benefit may be more enduring than that perceived by clinicians, highlighting the importance of shared decision making in the determination of retreatment timelines. Also of note, the proportion of participants who reported being satisfied with treatment was 53.9% at Day 90, despite marked improvements in both lower-facial width and participant-assessed MMP severity at this time point. This finding may suggest some participants desired a more dramatic effect on their lower-facial appearance than that achieved with a single treatment of onabotulinumtoxinA 72 U.

Treatment-related TEAEs reported during the DBPC period included mastication disorder (eg, masseter/jaw fatigue, masseter/jaw pain, chewing weakness; onabotulinumtoxinA, 3.5%; placebo, 0%) and injection-site pain (onabotulinumtoxinA, 2.8%; placebo, 2.2%). Treatment-related TEAEs were generally mild and resolved spontaneously. Reported TEAEs among onabotulinumtoxinA-treated participants are consistent with the onabotulinumtoxinA mechanism of action and injection location for treating MMP and were not unexpected, as previously reported in the literature evaluating onabotulinumtoxinA safety.^23^

Given the broad impact that MMP can have on the emotional, social, and professional aspects of individuals’ lives, understanding the holistic patient experience with this condition and its treatment is important for optimal care.^4-7^ The use of validated, condition-specific PRO measures complements conventional efficacy outcomes, such as lower-facial width. Condition-specific PRO data provide a comprehensive evaluation of individuals’ treatment experiences that can better inform clinicians in shared decision making with patients, and can enhance the drug development process by measuring treatment benefits most relevant to the target population. In addition to this strength, this study has some limitations. The study population was primarily female and Asian; therefore, the results may reflect some cultural nuances that would be worth further exploring in a more generalized population. Another potential limitation is the COVID-19 pandemic impact on study visits, as enrollment began in November 2019. A substantial proportion of participants discontinued (*n* = 52, 13.8%), and 28 participants (7.4%) enrolled in China and Taiwan at the start of the pandemic missed a study visit because of travel restrictions; after a pause, the authors of the study resumed enrollment in these regions once conduct was feasible. Finally, although ultrasound may be useful in the clinic setting to confirm the placement of dual-plane injections in the lower face, it was not utilized in this study.^24^

## CONCLUSIONS

In this pivotal Phase 3 study, a single treatment of onabotulinumtoxinA 72 U significantly improved the appearance and reduced the width of the lower face among participants with bilateral MMP for up to 6 months, maintaining an acceptable safety profile. Data from the validated PRO measures demonstrate high satisfaction, reduction in bother with the appearance of the lower face, and improvement in appearance-related psychosocial impact of MMP after onabotulinumtoxinA treatment.

## Supplemental Material

This article contains supplemental material located online at https://doi.org/10.1093/asj/sjaf204.

## Supplementary Material

sjaf204_Supplementary_Data
