# Supplementary material for: OnabotulinumtoxinA Treatment for Masseter Muscle Prominence: 6-Month Safety and Efficacy Results, Including Patient-Reported Outcomes, From a Phase 3, Randomized, Placebo-Controlled, Multiregional Trial
Source: Aesthet Surg J. 2025 Oct 15;46(5):486–94. doi: 10.1093/asj/sjaf204 (PMC13064654; doi:10.1093/asj/sjaf204)
Supplement: sjaf204_Supplementary_Data [file sjaf204_supplementary_data.zip › Supplemental Figure 1.docx]

**Supplemental Figure 1.** Study design. OnabotA, onabotulinumtoxinA.

^a^Screening period is up to 14 days prior to randomization on day 1.

^b^Re-treatment criteria: at least Grade 4 severity on each side by MMPS, not pregnant, at least 3 months since last treatment.
