# Supplementary material for: OnabotulinumtoxinA Treatment for Masseter Muscle Prominence: 6-Month Safety and Efficacy Results, Including Patient-Reported Outcomes, From a Phase 3, Randomized, Placebo-Controlled, Multiregional Trial
Source: Aesthet Surg J. 2025 Oct 15;46(5):486–94. doi: 10.1093/asj/sjaf204 (PMC13064654; doi:10.1093/asj/sjaf204)
Supplement: sjaf204_Supplementary_Data [file sjaf204_supplementary_data.zip › Supplemental Table 1.docx]

**Supplemental Table 1.** Study Instruments and Associated Endpoints

| **Instrument** | **Description** | **Associated Endpoint(s)** |
| --- | --- | --- |
| Masseter Muscle Prominence Scale (MMPS) | Investigator assessment of MMP severity on a 5-point scale ranging from 1 (“Minimal”) to 5 (“Very marked”) | 1. Achievement of ≥2-grade improvement from baseline at day 90 (primary endpoint)^a^ 2. Achievement of Grade ≤3 at day 90 (secondary endpoint)^a^ |
| Masseter Muscle Prominence Scale–Participant (MMPS-P) | Participant assessment of MMP severity on a 5-point scale from 1 (“Not at all pronounced” to 5 (“Very pronounced”) | 1. Achievement of ≥2-grade improvement from baseline at day 90 (secondary endpoint)^a^ 2. Achievement of Grade ≤3 at day 90 (secondary endpoint) |
| Participant Self-Assessment of Change (PSAC) | 1-item measure assessing change in lower face shape on a 7-point scale ranging from −3 (“Much worse”) to 3 (“Much improved”) | Achievement of PSAC Grade ≥1 (at least minimally improved from baseline) at day 90 (secondary endpoint)^a^ |
| Participant Global Impression of Bother (PGIB) | 1-item measure assessing bother related to the appearance of the lower face on a 5-point scale ranging from 0 (“Not at all bothered”) to 4 (“Extremely bothered”) | Responses of “Not at all bothered” or “A little bothered” among those who were at least “Somewhat bothered” at baseline |
| Lower Facial Shape Questionnaire (LFSQ) | | |
| Treatment Satisfaction Assessment (LFSQ-TXSAT: Follow-up version) | 1-item measure assessing satisfaction with the effect of treatment on the appearance of the lower face on a 5-point scale ranging from −2 (“Very dissatisfied”) to +2 (“Very satisfied”) | Responses of “Very satisfied” or “Satisfied” with MMP treatment |
| Impact Assessment (LFSQ-IA) | 6-item measure assessing psychosocial impact from the appearance of the lower face on a 5-point scale ranging from 0 (“Not at all”) to 4 (“Extremely”) or 0 (“Never”) to 4 (“Always”)  Summary score ranges from 0–24, where lower scores indicate less psychosocial impact | Mean change from baseline in summary score |

MMP, masseter muscle prominence.

^a^Achievement of ≥2-grade improvement in MMPS from baseline at day 90 was the predefined primary endpoint. At day 90, achievement of investigator-assessed MMPS or subject-assessed MMPS-P Grade ≤3, ≥2-grade improvement in MMPS-P, or achievement of Participant Self-Assessment of Change Grade ≥1 were predefined secondary endpoints. All other listed endpoints were exploratory.
