# Supplementary figures and images for: OnabotulinumtoxinA Treatment for Masseter Muscle Prominence: 6-Month Safety and Efficacy Results, Including Patient-Reported Outcomes, From a Phase 3, Randomized, Placebo-Controlled, Multiregional Trial
Source: Aesthet Surg J. 2025 Oct 15;46(5):486–94. doi: 10.1093/asj/sjaf204 (PMC13064654; doi:10.1093/asj/sjaf204)

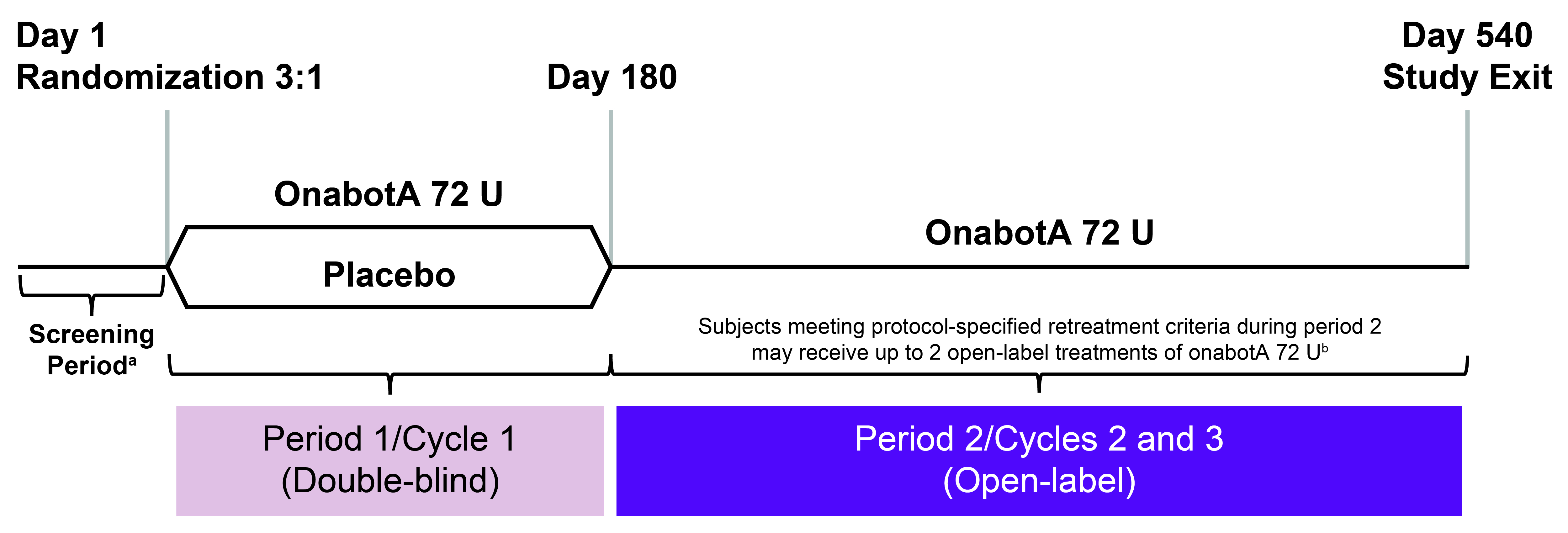

Supplement: sjaf204_Supplementary_Data [file sjaf204_supplementary_data.zip › Figure S1.jpg]
